# Joint large deviation result for empirical measures of the coloured random geometric graphs

**DOI:** 10.1186/s40064-016-2718-z

**Published:** 2016-07-20

**Authors:** Kwabena Doku-Amponsah

**Affiliations:** Statistics Department, University of Ghana, Box LG 115, Legon, Ghana

**Keywords:** Random geometric graph, Erdős–Rényi graph, Coloured random geometric graph, Typed graph, Joint large deviation principle, Empirical pair measure, Empirical measure, Degree distribution, Entropy, Relative entropy, Isolated vertices, 60F10, 05C80, 68P30

## Abstract

We prove joint large deviation principle for the * empirical pair measure* and *empirical locality measure* of the *near intermediate* coloured random geometric graph models on *n* points picked uniformly in a *d*-dimensional torus of a unit circumference. From this result we obtain large deviation principles for the *number of edges per vertex*, the *degree distribution and the proportion of isolated vertices * for the *near intermediate* random geometric graph models.

## Background

In this article we study the coloured geometric random graph CGRG, where *n* points or vertices or nodes are picked uniformly at random in $[0,1]^d,$ colours or spins are assigned independently from a finite alphabet $\Sigma$ and any two points with colours $a_1,a_2\in \Sigma$ distance at most $r_n(a_1,a_2)$ apart are connected. This random graph models, which has the geometric random graph (see Penrose 2003) as special case, has been suggested by Cannings and Penman (2003) as a possible extension to the coloured random graph studied in Biggins and Penman (2009), Doku-Amponsah and Mörters (2010), Doku-Amponsah (2006), Bordenave and Caputo (2015), Mukherjee (2014) and Doku-Amponsah (2014).

The connectivity radius $r_n$ plays similar role as the connection probability $p_n$ in the coloured random graph model. Several large deviation results about the coloured random graphs and hence Erdős–Rényi graph have been established recently. See O’Connell (1998), Biggins and Penman (2009), Doku-Amponsah and Mörters (2010), Doku-Amponsah (2006, 2014), Bordenave and Caputo (2015), Mukherjee (2014).

Until recently few or no large deviation result about the CGRG have been found. Doku-Amponsah (2015) proved joint large deviation principle for empirical pair measure and the empirical locality measure of the CGRG, where *n* points are uniformly chosen in $[0,1]^d$, colours or spins are assigned by drawing without replacement from the pool of, say, $n\nu _n(a_1)$ colours, and $n\omega _n(a_1,a_2)$ edges, $a_1,a_2\in \Sigma ,$ are randomly inserted among the points for some colour law $\nu _n{:}\;\Sigma \rightarrow [0,1]$ and edge law $\omega _n{:}\;\Sigma \times \Sigma \rightarrow [0,\infty ).$

This article presents a full joint large deviation principle (LDP) for the empirical pair measure and the empirical locality measure of the CGRG. Refer to (Doku-Amponsah and Mörters) for similar result for the coloured random graphs. From this large deviation results we obtain LDPs for graph quantities such as *number of edges per vertex, the degree distribution and the proportion of isolated vertices* of geometric random graphs in the intermediate case. Our results are similar to those in O’Connell (1998), Biggins and Penman (2009), Doku-Amponsah and Mörters (2010), Doku-Amponsah (2006, (2014), Bordenave and Caputo (2015) and Mukherjee (2014) for the Erdö–Renyi graph except that the rate functions of the LDPs in our current setting is bigger as a result of the effect of the geometric in the model.

As a first step in the proof of our main result, we obtain a joint LDP for the *empirical colour measure* and *empirical pair measures* for the CGRG, see Theorem 4, by the exponential change-of-measure techniques and coupling argument. See example Doku-Amponsah and Mörters (2010) or Doku-Amponsah (2006). In the next step, we use Biggins (2004, Theorem 5(b)) to mix Theorem 4 and the result (Doku-Amponsah 2015, Theorem 2.1) to obtain the full joint LDP for *empirical pair measure and the empirical locality measure* of CGRG model. Refer to Doku-Amponsah and Mörters (2010) or Doku-Amponsah (2006) for further illustration of this method.

Our main motivation for studying this model are in two folds.

### *Independence testing*

 Consider CGRG which is a model for Wireless Sensor Network as a very big dataset comprising the typed sites and the bonds between sites. One interesting question to ask is how many bits will be required to code the *n* sites and the bonds between sites with high probability? Then, an asymptotic equipartition property (AEP) for the WSN will answer this question and our LDP for the empirical measures of the CGRG will play a crucial in the prove of the AEP. Further, we can test whether a received codeword $y_n$ of WSN is jointly typical with a candidate sent codeword $x_n$ of WSN. The probability that two independent sequences $(x_n,y_n)$ ($x_n$ being a codeword other than what was sent when $y_n$ was received) actually appear as dependent is bounded asymptotically as $2^{-nI},$ where the AEP is used to obtain the bound. See Doku-Amponsah (2016) for more on this application.

### *Hypothesis testing*

 One of the standard problems in statistics is to decide between two alternative explanations for the data are observed. For example, a transmitter will send an information on the WSN bits by bits in communication systems. There are two possible cases for each transmission: one is that bit 0 of WSN data is sent (noted as event $H_0$) and the other is that bit 1 of WSN data is sent (noted as event $H_1$). In the receiver side, the bit *y* is to be received as either 0 or 1. Based on the *y* bit of WSN data received, we can make a hypothesis whether the event $H_0$ happens (bit 0 was sent at the transmitter) or the event $H_1$ happens (i.e. bit 1 was sent at the transmitter). Of course, we may make mis-judgement, such as we decode that bit 0 was sent but actually bit 1 was sent. We need to make the probability of error in hypothesis testing as low as possible and the LDPs for CGRG models can help us specify the probability of error.

In the remainder of the paper we state and prove our LDP results. In “Statement of the results” section we state our LDPs, Theorem 1, Corollary 2, Corollary 3, Theorem 4, and Corollary 5. In “Proof of Theorem 1” section we present the proof of Theorem 4. In “Proof of Theorem 1” section we combine Theorem 2.1 and Doku-Amponsah (2014, Theorem 2.1) to obtain the Theorem 1, using the setup and result of Biggins (2004) to ‘mix’ the LDPs. The paper concludes with the proofs of Corollaries 2, 3 and 5 which are given in “Proof of Corollaries 2, 3, and 5” section.

## Statement of the results

### The joint LDP for empirical pair measure and empirical locality measure of CGRG

In this subsection we shall look at a more general model of random geometric graphs, the CGCG in which the connectivity radius depends on the type or colour or symbol or spin of the nodes. The empirical pair measure and the empirical locality measure are our main object of study.

Given a probability measure $$\nu $$ on $$\Sigma $$ and a function $$r_n{:}\;\Sigma \times \Sigma \rightarrow (0,1]$$ we may define the *randomly coloured random geometric graph* or simply *coloured random geometric graph* $${\mathcal {G}}$$ with *n* vertices as follows: Pick vertices $$x_1,\ldots ,x_n$$ at random independently according to the uniform distribution on $$[0,\,1]^d,$$$$d\in \mathbb {N}.$$ Assign to each vertex $$x_j$$ colour $$\sigma (x_j)$$ independently according to the *colour law*$$\nu .$$ Given the colours, we join any two vertices $$x_i,x_j$$,$$(i\not =j)$$ by an edge independently of everything else, if

$$\begin{aligned} \Vert x_i-x_j\Vert \le r_n\left[ \sigma (x_i),\sigma (x_j)\right] . \end{aligned}$$

In this article we shall refer to $r_n(a,b),$ for $a,b\in \Sigma$ as a connection radius, and always consider

$$\begin{aligned} {\mathcal {G}}=(((\sigma (x_i),\sigma (x_j)){:}\;i,j=1,2,3,\ldots ,n),E) \end{aligned}$$

under the joint law of graph and colour. We interpret ${\mathcal {G}}$ as coloured GRG with vertices $x_1,\ldots ,x_n$ chosen at random uniformly and independently from the vertices space $[0,1]^2.$ For the purposes of this study we restrict ourselves to the near intermediate cases. i.e. the connection radius $r_n$ satisfies the condition $n r_n^d(a,b) \rightarrow C_d(a,b)$ for all $a,b\in \Sigma$, where $C_d{:}\;\Sigma ^2\rightarrow [0,\infty )$ is a symmetric function, which is not identically equal to zero.

For any finite or countable set $\Sigma$ we denote by ${\mathcal {P}}(\Sigma )$ the space of probability measures, and by $\tilde{\mathcal {P}}(\Sigma )$ the space of finite measures on $\Sigma$, both endowed with the weak topology. By convention we write $\mathbb {N}=\{0,1,2,\ldots \}.$

We associate with any coloured graph ${\mathcal {G}}$ a probability measure, the *empirical colour measure* ${\mathcal {L}}^1\in {\mathcal {P}}(\Sigma )$, by

$$\begin{aligned} {\mathcal {L}}_{{\mathcal {G}}}^{1}(a):=\frac{1}{n}\sum _{j=1}^{n}\delta _{\sigma (x_j)}(a),\quad \text{ for } a_1\in \Sigma , \end{aligned}$$

and a symmetric finite measure, the *empirical pair measure*${\mathcal {L}}_X^{2}\in \tilde{\mathcal {P}}_*(\Sigma ^2),$ by

$$\begin{aligned} {\mathcal {L}}_{{\mathcal {G}}}^{2}(a,b):=\frac{1}{n}\sum _{(i,j)\in E}[\delta _{(\sigma (x_i),\sigma (x_j))}+ \delta _{((\sigma (x_j),\sigma (x_i))}](a,b),\quad \text{ for } (a, b)\in \Sigma ^2. \end{aligned}$$

Note that the total mass the empirical pair measure is 2|*E*| / *n*. Finally we define a further probability measure, the *empirical neighbourhood measure*${\mathcal {M}}_{{\mathcal {G}}}\in {\mathcal {P}}(\Sigma \times \mathbb {N})$, by

$$\begin{aligned} {\mathcal {M}}_{{\mathcal {G}}}(a,\ell ):=\frac{1}{n}\sum _{j=1}^{n}\delta _{(\sigma (x_i),L(x_j))}(a,\ell ),\quad \text{ for } (a,\ell )\in \Sigma \times \mathbb {N}, \end{aligned}$$

while $L(x_j)=(l^{x_j}(b),\,b\in \Sigma )$ and $l^{x_j}(b)$ is the number of vertices of colour *b* connected to vertex $x_j$.

For any $\eta \in {\mathcal {P}}(\Sigma \times \mathbb {N}^{\Sigma })$we denote by $\eta _1$ the $\Sigma$-marginal of $\eta$ and for every $(b,a)\in \Sigma \times \Sigma ,$ let $\eta _2$ be the law of the pair (*a*, *l*(*b*)) under the measure $\eta .$ Define the measure (finite), $\langle \eta (\cdot ,\ell ),\,l(\cdot )\rangle \in \tilde{\mathcal {P}}(\Sigma \times \Sigma )$ by

$$\begin{aligned} {\mathcal {H}}_2(\eta )(b,a):= \sum _{l(b)\in \mathbb {N}}\eta _2(a,l(b))l(b), \quad \text{ for } a,b\in \Sigma \end{aligned}$$

and write ${\mathcal {H}}_1(\eta )=\eta _1.$ We define the function ${\mathcal {H}}{:}\; {\mathcal {P}}(\Sigma \times \mathbb {N}^{\Sigma }) \rightarrow {\mathcal {P}}(\Sigma ) \times \tilde{\mathcal {P}}(\Sigma \times \Sigma )$ by ${\mathcal {H}}(\eta )=({\mathcal {H}}_1(\eta ),{\mathcal {H}}_2(\eta ))$ and note that ${\mathcal {H}}({\mathcal {M}}_{{\mathcal {G}}})=({\mathcal {L}}_{{\mathcal {G}}}^1, {\mathcal {L}}_{{\mathcal {G}}}^2).$ Observe that ${\mathcal {H}}_1$ is a continuous function but ${\mathcal {H}}_2$ is *discontinuous* in the weak topology. In particular, in the summation $\sum \nolimits _{l(b)\in \mathbb {N}}\eta _2(a,l(b))l(b)$ the function *l*(*b*) may be unbounded and so the functional $\displaystyle \eta \rightarrow {\mathcal {H}}_2(\eta )$ would not be continuous in the weak topology. We call a pair of measures $(\omega , \eta )\in \tilde{{\mathcal {P}}}(\Sigma \times \Sigma )\times {\mathcal {P}}(\Sigma \times \mathbb {N}^{\Sigma })$*sub-consistent* if

1 $$\begin{aligned} {\mathcal {H}}_2(\eta )(b,a) \le \omega (b,a), \quad \text{ for } \text{ all } a,b\in \Sigma , \end{aligned}$$

and *consistent* if equality holds in (1). For a measure $\omega \in \tilde{\mathcal {P}}_*(\Sigma ^2)$ and a measure $\rho \in {\mathcal {P}}(\Sigma )$, we recall from (Doku-Amponsah and Mörters 2010) the rate function

$$\begin{aligned} {{\mathfrak {H}}}_1(\omega \, \Vert \, \rho ):= H\left( \omega \,\Vert \,C_d\rho \otimes \rho \right) +\Vert C_d\rho \otimes \rho \Vert -\Vert \omega \Vert , \end{aligned}$$

where the measure $C_d\rho \otimes \rho \in \tilde{\mathcal {P}}(\Sigma \times \Sigma )$ is defined by $C_d\rho \otimes \rho (a,b)=C_d(a,b)\rho (a)\rho (b)$ for $a,b\in \Sigma$. It is not hard to see that ${\mathfrak {H}}_1(\omega \,\Vert \,\rho )\ge 0$ and equality holds if and only if $\omega = C_d\rho \otimes \rho$.

For every $(\omega ,\eta )\in \tilde{\mathcal {P}}_*(\Sigma \times \Sigma ) \times {\mathcal {P}}(\Sigma \times \mathbb {N})$ define a probability measure $Q_{poi}^{(\omega ,\eta )}$ on $\Sigma \times \mathbb {N}$ by

$$\begin{aligned} Q_{poi}^{(\omega ,\eta )}(a,\ell ):=\eta _{1}(a)\prod _{b\in \Sigma } e^{-\frac{\omega (a,b)}{\eta _1(a)}} \, \frac{1}{\ell (b)!}\, \left( \frac{\omega (a,b)}{\eta _1(a)}\right) ^{\ell (b)}, \quad \text{ for } a\in \Sigma , \ell \in \mathbb {N}. \end{aligned}$$

We assume $d\in \mathbb {N}$ and write

$$\begin{aligned} \begin{aligned} \Delta (d)=\left\{ \begin{array}{ll} \frac{\pi ^{d/2}}{\Gamma \left( \frac{(d+2)}{2}\right) }&{} \quad \text{ if } d\ge 2\\ \mathbbm {1}&{} \quad \text{ if } d=1, \end{array}\right. \end{aligned} \end{aligned}$$

where $\Gamma$ is the gamma function. We now state the principal theorem in this section the LDP for the empirical pair measure and the empirical locality measure.

#### **Theorem 1**

*Suppose that*${\mathcal {G}}$*is a CRGG with colour law*$\nu$*andconnection radii*$r_n{:}\;\Sigma \times \Sigma \rightarrow [0,1]$*satisfying*$n r_n^d(a,b) \rightarrow C_d(a,b)$*for some symmetric function*$C{:}\;\Sigma \times \Sigma \rightarrow [0,\infty )$*not identical tozero. Then, as*$n\rightarrow \infty ,$*the pair*$({\mathcal {L}}_{{\mathcal {G}}}^2,\,{\mathcal {M}}_{{\mathcal {G}}})$*satisfies an LDP in*$\tilde{{\mathcal {P}}}_*(\Sigma \times \Sigma )\times {\mathcal {P}}(\Sigma \times \mathbb {N})$*with good rate function*

$$\begin{aligned}&\begin{aligned}&J(\omega ,\eta )=\left\{ \begin{array}{ll}H(\eta \,\Vert \,Q_{poi}^{(\omega ,\eta )})+H(\eta _1\,\Vert \,\nu )+\frac{1}{2}{{\mathfrak {H}}}_2(\omega \Vert \eta _1)&{}\quad  if\; (\omega ,\eta )\;consistent \; and \;\eta _1=\omega _2,\\ \infty &{} \quad otherwise.  \end{array}\right. \end{aligned}\\&{{\mathfrak {H}}}_2(\omega \Vert \eta _1)= \, {{\mathfrak {H}}}_1(\omega \,\Vert \,\eta _1 )-\Vert \omega \Vert \log \Delta (d)+(\Delta (d)-\mathbbm {1})\Vert C_d \eta _1\otimes \eta _1\Vert . \end{aligned}$$

#### *Remark 1*

Note that the first three terms of the rate function is the same as the rate function of Doku-Amponsah and Mörters (2010, Theorem 2.1). Additionally, the extra term $$\frac{1}{2}( -\Vert \omega \Vert \log \Delta (d)+(\Delta (d)-\mathbbm {1})\Vert C_d \eta _1\otimes \eta _1\Vert )$$ is positive and is as a result of the geometric $$[0,\,1]^d$$ we have incorporated in the model. Moreover, on typical CGRG we have, $$\eta _1=\nu ,$$$$\omega = \Delta (d) C \,\eta _1 \otimes \eta _1$$ and

$$\begin{aligned} \eta (a,\ell )=\nu (a)\prod _{b\in \Sigma }e^{-\Delta (d) C_d(a,b)\nu (b)}\, \frac{(\Delta (d)C_d(a,b)\nu (b))^{\ell (b)}}{\ell (b)!},\quad \text{ for } \text{ all } (a,\ell )\in \Sigma \times \mathbb {N}. \end{aligned}$$

Hence, for some $\varepsilon$ we $\mathbb {P}\left\{ |{\mathcal {M}}_{{\mathcal {G}}}-\eta \Vert \ge \varepsilon \right\} \rightarrow 0$ as $n\rightarrow \infty .$

We write

$$\begin{aligned} \lambda _1(\delta ):=\left( \Delta (d)- \mathbbm {1} \right) \frac{c}{2}- \frac{1}{2} \, \langle \delta \rangle \, \log \Delta (d) \end{aligned}$$

#### **Corollary 2**

*SupposeDis the degree distribution of the random graph*${\mathcal {G}}(n,r_n),$*where the connectivity radius*$r_n\in (0,1]$*satisfies*$n r_n^d \rightarrow c \in (0,\infty )$. *Then, as*$n\rightarrow \infty$, *Dsatisfies an LDP in the space*${\mathcal {P}}(\mathbb {N}\cup \{0\})$*with good rate function*

2 $$\begin{aligned} \begin{aligned} \lambda _2(\delta )= \left\{ \begin{array}{ll}\left[ H (d\,\Vert \,q_{\langle \delta \rangle })+\frac{1}{2} \, \langle \delta \rangle \, \log \left( \frac{\langle \delta \rangle }{c} \right) - \frac{1}{2} \, \langle \delta \rangle + \frac{c}{2}\right] + \lambda _1(\delta ), &{} \quad \text{ if } \langle \delta \rangle < \infty ,\\ \infty &{} \quad \text{ if } \langle \delta \rangle = \infty , \end{array}\right. \end{aligned} \end{aligned}$$

*where*$q_{k}$*is a poisson distribution with parameter* *k*,  *and*$\langle \delta \rangle := \sum _{m=0}^{\infty }m\delta (m)$.

This rate function $\lambda _2$ compares very well with the rate function of Doku-Amponsah and Mörters (2010, Corollary 2.2) with the extra term $\lambda _1$ accounting for the geometric effect on the CGRG model.

Next we give a similar result as in O’Connell (1998), the LDP for the proportion of isolated vertices of the RGG.

$$\begin{aligned} \xi _1(y) =( \Delta (d)-\mathbbm {1}) c y(1-y/2) + (1-y) \left[ \log \left( \frac{\mathbbm {1}}{\Delta (d)}\right) - \frac{(\Delta (d)-\mathbbm {1})c (1-y))}{2} \right] \end{aligned}$$

#### **Corollary 3**

*SupposeDis the degree distribution of the random graph*${\mathcal {G}}(n,r_n),$*where the connectivity radius*$r_n\in (0,1]$*satisfies*$n r_n^d \rightarrow c \in (0,\infty )$. *Then, as*$n\rightarrow \infty$, *the proportion of isolated vertices*, *D*(0) *satisfies an LDP in* [0, 1] *with good rate function*

$$\begin{aligned} \xi _2(y)=y \log y + c y(1-y/2) - (1-y) \left[ \log \left( \frac{c}{a}\right) - \frac{(a- c (1-y))^2}{2c(1-y)} \right] +\xi _1(y) , \end{aligned}$$

*where*$a=a(y,d)$*is the unique positive solution of*$1-e^{-a}=\frac{\Delta (d)c}{a}\, (1-y)$.

From Corollary 3 we deduce that on a typical random geometric graphs the number of isolated vertices will grow like $n e^{-\Delta (d)c}.$ Thus, as $n\rightarrow \infty ,$ the number of isolated vertices in the geometric random graphs converges to $n e^{-\Delta (d)c}$ in probability. Again, the rate function $\xi _2$ above compares very well with the result of O’Connell (1998) with the extra term $\xi _1$ accounting for the influence of the geometric plane $[0, 1]^d$ on the model.

### The joint LDP for the empirical colour measure and empirical pair measure of CGRG

#### **Theorem 4**

*Suppose that*${\mathcal {G}}$*is a CGRG with colour law*$\nu$*and connection radii*$r_n{:}\;\Sigma ^2\rightarrow [0,1]$*satisfying*$n r_n^d(a,b) \rightarrow C_d(a,b)$*for some symmetric function*$C_d{:}\;\Sigma ^2\rightarrow [0,\infty )$*not identical to zero. Then, as*$n\rightarrow \infty ,$*the pair*$({\mathcal {L}}_X^1,{\mathcal {L}}_X^2)$*satisfies an LDP in*${\mathcal {P}}(\Sigma )\times \tilde{{\mathcal {P}}}_*(\Sigma ^2)$*with good rate function*

3 $$\begin{aligned} I(\eta _1,\omega )=H(\eta _1\,\Vert \,\nu )+\frac{1}{2}{{\mathfrak {H}}}_{2}(\omega \,\Vert \,\eta _1 ),\, \end{aligned}$$

*where the measure*$C\eta _1\otimes \eta _1 \in \tilde{\mathcal {P}}_*(\Sigma \times \Sigma )$*is defined by*$C\eta _1\otimes \eta _1 (a,b)=C_d(a,b)\eta _1(a)\eta _1(b)$*for*$a,b\in \Sigma .$

Further, we state a corollary of Theorem 4 below.

#### **Corollary 5**

*Suppose that*${\mathcal {G}}\hbox {is}$*a CGRG graph with colour law*$\nu$*and connection radii*$r_n{:}\;\Sigma ^2\rightarrow [0,1]$*satisfying*$n r_n^d(a,b) \rightarrow C_d(a,b)$*for some symmetric function*$C_d{:}\;\Sigma ^2\rightarrow [0,\infty )$*not identical to zero. Then, as*$n\rightarrow \infty ,$*the number of edges per vertex* |*E*| / *nof*${\mathcal {G}}\hbox {satisfies}$*an LDP in*$[0,\infty )$*with good ratefunction*

$$\begin{aligned} \zeta (x)= x \log x - x+ \inf _{y>0} \left\{ \psi (y) - x \log (y) + y \right\} , \end{aligned}$$

*where*$\psi (y) = \inf H(\eta _1 \, \Vert \, \nu )$*over all probabilityvectors*$\eta _1$*with*$\frac{1}{2} \Delta (d)\eta _1^{T} C \eta _1 =y$.

#### *Remark 2*

By taking $C_d(a,b)=c$ one will obtain $\psi (y)=0$ for $y=\frac{\Delta (d) }{2}c$, and $\psi (y)=\infty$ otherwise, which establishes that |*E*| / *n* obeys an LDP in $[0,\infty )$ with good rate function

$$\begin{aligned} \zeta (x)= x \log x - x+ \inf _{y>0} \left\{ \psi (y) - x \log \left( \frac{1}{2} y\right) + \frac{1}{2} y \right\} , \end{aligned}$$

where $$\Delta (d)c =y$$.

## Proof of Theorem 4

### Change-of-measure

For any two points $U_1$ and $U_2$ uniformly and independently chosen from the space $[0,\,1]^d$ write

$$\begin{aligned} F(t):=\mathbb {P}\left\{ \Vert U_1-U_2\Vert \le t\right\} . \end{aligned}$$

Further, given a function $\tilde{f}{:}\;\Sigma \rightarrow \mathbb {R}$ and a symmetric function $\tilde{g}{:}\; \Sigma ^2\rightarrow \mathbb {R}$, we define the constant $U_{\tilde{f}}$ by

$$\begin{aligned} U_{\tilde{f}}=\log \sum _{a\in \Sigma }e^{\tilde{f}(a)}\nu (a), \end{aligned}$$

and the function $\tilde{h}_n{:}\;\Sigma ^2\rightarrow \mathbb {R}$ by

4 $$\begin{aligned} \tilde{h}_n(a,b)=\log \left[ \left( 1-F(r_n(a,b))+ F(r_n(a,b)) e^{\tilde{g}(a,b)}\right) ^{-n}\right] , \end{aligned}$$

for $a,b\in \Sigma .$ We use $\tilde{f}$ and $\tilde{g}$ to define (for sufficiently large *n*) a new coloured random graph as follows:

- To the *n* points $x_1,x_2,\ldots ,x_n$ picked independently and uniformly in $[0,1]^d$ we assign colours from $\Sigma$ independently and identically according to the colour law $\tilde{\nu }$ defined by
  $$\begin{aligned} \tilde{\nu }(a)=e^{\tilde{f}(a)-B_{\tilde{f}}}\nu (a). \end{aligned}$$
- Given any two points $x_u,x_v,$ with $x_u$ carrying colour *a* and $x_v$ carrying colour *b*, we connect vertex $x_u$ to vertex $x_v$ with probability
  $$\begin{aligned} F(\tilde{r}_{n}(a,b))=\frac{F(r_n(a,b))e^{\tilde{g}(a,b)}}{1-F(r_n(a,b))+F(r_n(a,b))e^{\tilde{g}(a,b)}}. \end{aligned}$$

We denote the transformed law by $\tilde{\mathbb {P}}.$ We observe that $\tilde{\nu }$ is a probability measure and that $\tilde{\mathbb {P}}$ is absolutely continuous with respect to $\mathbb {P}$ as, for any coloured graph ${\mathcal {G}}=((\sigma (x_j){:}\; j=1,2,3,\ldots ,n),E)$,

5 $$\begin{aligned} \frac{d\tilde{\mathbb {P}}}{d\mathbb {P}}({\mathcal {G}})&= \prod _{u\in V}\frac{\tilde{\nu }(\sigma (x_u))}{\nu (\sigma (x_u))}\prod _{(u,v)\in E}\frac{F(\tilde{r}_{n}(\sigma (x_u),\sigma (x_v)))}{F(r_n(\sigma (x_u),\sigma (x_v)))}\prod _{(u,v)\not \in E}\frac{1-F(\tilde{r}_n(\sigma (x_u),\sigma (x_v)))}{1-F(r_n(\sigma (x_u),\sigma (x_v)))}\nonumber \\&= \prod _{u\in V}\frac{\tilde{\nu }(\sigma (x_u))}{\nu (\sigma (x_u))}\prod _{(u,v)\in E}\frac{F(\tilde{r}_n(\sigma (x_u),\sigma (x_v)))}{F(r_n(\sigma (x_u),\sigma (x_v)))} \times \frac{n-nF(r_n(\sigma (x_u),\sigma (x_v)))}{n-nF(\tilde{r}_n(\sigma (x_u),\sigma (x_v)))}\\&\quad\times\prod _{(u,v)\in {\mathcal {E}}}\frac{n-nF(\tilde{r}_n(\sigma (x_u),\sigma (x_v)))}{n-nF(r_n(\sigma (x_u),\sigma (x_v)))}\nonumber \\&= \prod _{u\in V}e^{\tilde{f}(\sigma (x_u))-U_{\tilde{f}}}\prod _{(u,v)\in E}e^{\tilde{g}(\sigma (x_u),\sigma (x_v))}\prod _{(u,v)\in {\mathcal {E}}}{e^{\frac{1}{n}\, \tilde{h}_n(\sigma (x_u),\sigma (x_v))}}\nonumber \\&= \exp \left( n\left\langle {\mathcal {L}}_{{\mathcal {G}}}^1, \tilde{f}-U_{\tilde{f}}\right\rangle +n\left\langle \frac{1}{2}{\mathcal {L}}_{{\mathcal {G}}}^2, \tilde{g}\right\rangle +n\left\langle \frac{1}{2}{\mathcal {L}}_{{\mathcal {G}}}^1\otimes {\mathcal {L}}_{{\mathcal {G}}}^1, \tilde{h}_n\right\rangle -\left\langle \frac{1}{2}L_{\Delta }^{1}, \tilde{h}_n\right\rangle \right) , \end{aligned}$$

where

$$\begin{aligned} L_{\Delta }^{1}=\frac{1}{n}\sum _{u\in V}\delta _{(\sigma (x_u),\sigma (x_u))}. \end{aligned}$$

We write $\langle g,\omega \rangle :=\sum _{a,b\in \Sigma } g(a,b)\omega (a,b)$ for $\omega \in \tilde{{\mathcal {P}}}(\Sigma ^2)$, and $\langle f,\rho \rangle :=\sum _{a\in \Sigma } f(a)\rho (a)$ for $\rho \in {\mathcal {P}}(\Sigma )$, and note that

$$\begin{aligned} F(r_n(a,b))=\Delta (d)r_n^d(a,b),\quad \text{for } \text{ all } a,b\in \Sigma ^2. \end{aligned}$$

i.e. the volume of a *d*-dimensional (hyper)sphere with radius *r*(*a*, *b*) satisfying $nr_n^d (a,b)\rightarrow C_d(a,b).$

The following lemmas will be useful in the proofs of main Lemmas.

#### **Lemma 1**

(Euler’s lemma) *If*$nr_n^d (a,b)\rightarrow C_d(a,b)$*for every*$a,b\in \Sigma$, *then*

6 $$\begin{aligned} \lim _{n\rightarrow \infty }\left[ 1+\alpha F(r_n(a,b))\right] ^{n}=e^{\alpha \Delta (d) C_d(a,b)},\quad \text{ for } \text{ all } a,b\in \Sigma \text{ and } \alpha \in \mathbb {R}. \end{aligned}$$

#### *Proof*

Observe that, for any $\varepsilon >0$ and for large *n* we have

$$\begin{aligned} \left[ 1+\frac{\alpha \Delta (d)C_d(a,b)-\varepsilon }{n}\right] ^{n}\le \left[ 1+ \alpha F(r_n(a,b))\right] ^{n}\le \left[ 1+\frac{\alpha \Delta (d) C_d(a,b)+\varepsilon }{n}\right] ^{n}, \end{aligned}$$

by the point-wise convergence. Hence by the sandwich theorem and Euler’s formula we get (6). $\square$

We write

$$\begin{aligned} P^{(n)}(\omega ) := \mathbb {P}\left\{ {\mathcal {L}}_{{\mathcal {G}}}^1=\omega \right\} . \end{aligned}$$

#### **Lemma 2**

*The family of measures*$({P}^n {:}\; n\in \mathbb {N})$*is exponentially tight on*${\mathcal {P}}(\Sigma )$.

#### *Proof*

We use coupling argument, see the proof of Doku-Amponsah and Mörters (2010, Lemma 5.1) to show that, for every $\theta >0$, there exists $N\in \mathbb {N}$ such that

$$\begin{aligned} \limsup _{n\rightarrow \infty }\frac{1}{n}\mathbb {P}\left\{ |E|>nN\right\} \le -\theta . \end{aligned}$$

To begin, let $c(d)>\max _{a,b\in \Sigma }C_d(a,b)>0$ and $nr_n^d(c)\rightarrow c(d).$ Using similar coupling arguments as in see the proof of Doku-Amponsah and Mörters (2010, Lemma 5.1), we can define, for all sufficiently large *n*,  a coloured random graph $\tilde{X}$ with vertices $x_1,\ldots ,x_n$ chosen uniformly from the vertices space $[0, 1]^d,$ colour law $\eta$ and connectivity probability $p_n=\mathbb {P}\left\{ \Vert x_i-x_j\Vert \le r_n(c)\right\} = \Delta (d)r_n^d,$ for all $i\not =j$ such that any edge present in ${\mathcal {G}}$ is also present in $\tilde{X}.$ Let $|\tilde{E}|$ be the number of edges of $\tilde{X}.$ Using the binomial formula and Euler’s formula, we have that

$$\begin{aligned} \begin{aligned} \mathbb {P}\{ |\tilde{E}|\ge n l\}\le e^{-nl}\mathbb {E}\left[ e^{|\tilde{E}|}\right]&=e^{-nl}\sum _{k=0}^{\frac{n(n-1)}{2}}e^{k} \left( \genfrac{}{}{0.0pt}{}{n(n-1)/2}{ k}\right) \left( p_n\right) ^{k}\left( 1-p_n\right) ^{n(n-1)/2-k}\\&= e^{-nl}\left( 1-p_n+ep_n\right) ^{n(n-1)/2} \le e^{-nl}e^{nc\Delta (d)(e-1+o(1))}, \end{aligned} \end{aligned}$$

where we used $np_n=\Delta (d)nr_n^d\rightarrow \Delta (d)c$ in the last step. Now given $\theta >0$ choose $N\in \mathbb {N}$ such that $N> \theta +\Delta (d)c(e-1)$ and observe that, for sufficiently large *n*, 

$$\begin{aligned} \mathbb {P}\left\{ |E|\ge n N\right\} \le \mathbb {P}\{ |\tilde{E}|\ge nN\}\le e^{-n\theta }, \end{aligned}$$

which implies the statement. $\square$

### Proof of the upper bound in Theorem 4

We denote by ${\mathcal {C}}_1$ the space of functions on $\Sigma$ and by ${\mathcal {C}}_2$ the space of symmetric functions on $\Sigma ^2$, and define

$$\begin{aligned} \hat{I}({\eta }_{1},\omega )=\sup _{\genfrac{}{}{0.0pt}{}{f\in {\mathcal {C}}_{1}}{g\in {\mathcal {C}}_{2}}} \left\{ \sum _{a\in \Sigma }\left( f(a)-U_{f}\right) \eta _{1}(a) +\frac{1}{2} \sum _{a,b\in \Sigma }g(a,b)\omega (a,b)+ \frac{\Delta (d)}{2} \sum _{a,b\in \Sigma }(1-e^{g(a,b)})C_d(a,b)\eta _{1}(a) \eta _{1}(b) \right\} \end{aligned}$$

for $$({\eta }_{1},\omega )\in {\mathcal {P}}(\Sigma )\times {\mathcal {P}}_*(\Sigma ^2)$$

#### **Lemma 3**

*For each closed set*$G\subset {\mathcal {P}}(\Sigma )\times \tilde{{\mathcal {P}}}_*(\Sigma ^2),$*we have*

$$\begin{aligned} \limsup _{n\rightarrow \infty }\frac{1}{n}\log \mathbb {P}\left\{ \left( {\mathcal {L}}_{{\mathcal {G}}}^1,{\mathcal {L}}_{{\mathcal {G}}}^2\right) \in F\right\} \le -\inf _{({\eta }_{1},\omega )\in F}\hat{I}({\eta }_{1},\omega ). \end{aligned}$$

#### *Proof*

First let $\tilde{f}\in {\mathcal {C}}_1$ and $\tilde{g}\in {\mathcal {C}}_2$ be arbitrary. Define $\tilde{\beta }{:}\;\Sigma ^2\rightarrow \mathbb {R}$ by

$$\begin{aligned} \tilde{\beta }(a,b)=\Delta (d)(1-e^{\tilde{g}(a,b)})C_d(a,b). \end{aligned}$$

Observe that, by Lemma 1, $\tilde{\beta }(a,b)=\lim _{n\rightarrow \infty }\tilde{h}_n(a,b)$ for all $a,b\in \Sigma$, recalling the definition of $\tilde{h}_n$ from (4). Hence, by (5), for sufficiently large *n*,

$$\begin{aligned} e^{\max _{a\in \Sigma } | \tilde{\beta }(a,a)| } \ge \int e^{\langle \frac{1}{2}L_{\Delta }^{1},\, \tilde{h}_n\rangle }d\tilde{\mathbb {P}} =\mathbb {E}\left\{ e^{n\langle {\mathcal {L}}_{{\mathcal {G}}}^1, \tilde{f}-U_{\tilde{f}}\rangle +n\langle \frac{1}{2}{\mathcal {L}}_{{\mathcal {G}}}^2, \tilde{g}\rangle +n\langle \frac{1}{2}{\mathcal {L}}_{{\mathcal {G}}}^1\otimes {\mathcal {L}}_{{\mathcal {G}}}^1, \tilde{h}_n\rangle }\right\} , \end{aligned}$$

where $L_{\Delta }^{1}=\frac{1}{n}\sum _{u\in V}\delta _{(\sigma (x_u),\sigma (x_u))}$ and therefore,

7 $$\begin{aligned} \limsup _{n\rightarrow \infty }\frac{1}{n}\log \mathbb {E}\left\{ e^{n\left\langle {\mathcal {L}}_{{\mathcal {G}}}^1,\tilde{f}-U_{\tilde{f}}\right\rangle +n\left\langle \frac{1}{2}{\mathcal {L}}_{{\mathcal {G}}}^2, \tilde{g}\right\rangle +n\left\langle \frac{1}{2}\,{\mathcal {L}}_{{\mathcal {G}}}^1\otimes {\mathcal {L}}_{{\mathcal {G}}}^1 , \tilde{h}_n\right\rangle }\right\} \le 0. \end{aligned}$$

Given $\varepsilon >0$ let $\hat{I}_{\varepsilon }({\eta }_{1},\omega )=\min \{\hat{I}({\eta }_{1},\omega ),{\varepsilon }^{-1}\}-\varepsilon .$ Suppose that $({\eta }_{1},\omega )\in G$ and observe that $\hat{I}({\eta }_{1},\omega )>\hat{I}_{\varepsilon }({\eta }_{1},\omega ).$ We now fix $\tilde{f}\in {\mathcal {C}}_1$ and $\tilde{g}\in {\mathcal {C}}_2$ such that

$$\begin{aligned} \langle \tilde{f}-U_{\tilde{f}},\eta _1\rangle + \frac{1}{2}\,\langle \tilde{g},\omega \rangle + \frac{1}{2} \, \langle \tilde{\beta },\eta _1\otimes \eta _1 \rangle \ge \hat{I}_{\varepsilon }({\eta }_{1},\omega ) . \end{aligned}$$

As $\Sigma$ is finite, there exist open neighbourhoods $B_{\eta _1}^{1}$ and $B_{\omega }^{2}$ of ${\eta }_{1},\omega$ such that

$$\begin{aligned} \inf _{\genfrac{}{}{0.0pt}{}{\tilde{\eta }_1\in B_{\eta _1}^{1}}{\tilde{\omega }\in B_{\omega }^{2}}}\left\{ \langle \tilde{f}-U_{\tilde{f}} , \eta _1\rangle + \frac{1}{2} \, \langle \tilde{g},\tilde{\omega }\rangle +\frac{1}{2} \, \langle \tilde{\beta },\eta _1\otimes \eta _1\rangle \right\} \ge \hat{I}_{\varepsilon }({\eta }_{1},\omega )-\varepsilon . \end{aligned}$$

Using Chebyshev’s inequality and (7) we have that

8 $$\begin{aligned} \begin{aligned}&\limsup _{n\rightarrow \infty }\frac{1}{n} \log \mathbb {P}\left\{ \left( {\mathcal {L}}_{{\mathcal {G}}}^1, {\mathcal {L}}_{{\mathcal {G}}}^2\right) \in B_{\eta _1}^{1}\times B_{\omega }^{2}\right\} \\&\quad \le \limsup _{n\rightarrow \infty }\frac{1}{n}\log \mathbb {E}\left\{ e^{n\left\langle {\mathcal {L}}_{{\mathcal {G}}}^1,\tilde{f}-U_{\tilde{f}}\right\rangle +n\left\langle \frac{1}{2}{\mathcal {L}}_{{\mathcal {G}}}^2,\tilde{g}\right\rangle +n\left\langle \frac{1}{2}{\mathcal {L}}_{{\mathcal {G}}}^1\otimes {\mathcal {L}}_{{\mathcal {G}}}^1,\tilde{h}_n\right\rangle }\right\} -\hat{I}_{\varepsilon }({\eta }_{1},\omega )+\varepsilon \\&\quad \le -\hat{I}_{\varepsilon }({\eta }_{1},\omega )+\varepsilon . \end{aligned} \end{aligned}$$

Now we use Lemma 2 with $\theta =\varepsilon ^{-1},$ to choose $N(\varepsilon )\in \mathbb {N}$ such that

9 $$\begin{aligned} \limsup _{n\rightarrow \infty }\frac{1}{n}\log \mathbb {P}\left\{ |E|> n N(\varepsilon ) \right\} \le -\varepsilon ^{-1}. \end{aligned}$$

For this $N(\varepsilon ),$ define the set $K_{N(\varepsilon )}$ by

$$\begin{aligned} K_{N(\varepsilon )}=\left\{ ({\eta }_{1},\omega )\in {\mathcal {P}}(\Sigma )\times \tilde{{\mathcal {P}}}_*(\Sigma ^2){:}\;\Vert \omega \Vert \le 2N(\varepsilon )\right\} , \end{aligned}$$

and recall that $\Vert {\mathcal {L}}_{{\mathcal {G}}}^2\Vert =2{|E|}/{n}.$ The set $K_{N(\varepsilon )}\cap F$ is compact and therefore may be covered by finitely many sets $B_{\eta _{1,r}}^{1}\times B_{\omega _ {r}}^{2},r=1,\ldots ,m$ with $(\eta _{1,r},\omega _{r})\in F$ for $r=1,\ldots ,m.$ Consequently,

$$\begin{aligned} \mathbb {P}\left\{ \left( {\mathcal {L}}_{{\mathcal {G}}}^1, {\mathcal {L}}_{{\mathcal {G}}}^2\right) \in F\right\} \le \sum _{r=1}^{m}\mathbb {P}\left\{ \left( {\mathcal {L}}_{{\mathcal {G}}}^1, {\mathcal {L}}_{{\mathcal {G}}}^2\right) \in B_{\eta _{1,r}}^{1}\times B_{\omega _{r}}^{2}\right\} +\mathbb {P}\left\{ \left( {\mathcal {L}}_{{\mathcal {G}}}^1, {\mathcal {L}}_{{\mathcal {G}}}^2\right) \not \in K_{N(\varepsilon )}\right\} . \end{aligned}$$

We may now use (8) and (9) to obtain, for all sufficiently small $\varepsilon >0$,

$$\begin{aligned} \begin{aligned} \limsup _{n\rightarrow \infty }\frac{1}{n}\log \mathbb {P}\left\{ \left( {\mathcal {L}}_{{\mathcal {G}}}^1, {\mathcal {L}}_{{\mathcal {G}}}^2\right) \in F\right\}&\le \max _{r=1}^{m} \left( \limsup _{n\rightarrow \infty }\frac{1}{n}\log \mathbb {P}\left\{ \left( {\mathcal {L}}_{{\mathcal {G}}}^1, {\mathcal {L}}_{{\mathcal {G}}}^2\right) \in B_{\eta _{1,r}}^{1}\times B_{\omega _{r}}^{2}\right\} \right) \vee (-\varepsilon )^{-1} \\&\le \left( -\inf _{({\eta }_{1},\omega )\in G}\hat{I}_{\varepsilon }({\eta }_{1},\omega )+\varepsilon \right) \vee (-\varepsilon )^{-1}. \end{aligned} \end{aligned}$$

Taking $\varepsilon \downarrow 0$ we get the desired statement. $\square$

Next, we express the rate function in term of relative entropies, see for example Dembo and Zeitouni (1998, 2.15), and consequently show that it is a good rate function. Recall the definition of the function *I* from Theorem 4.

#### **Lemma 4**

(i) $$\hat{I}({\eta }_{1},\omega )=I({\eta }_{1},\omega ),$$*for any*$$({\eta }_{1},\omega )\in {\mathcal {P}}(\Sigma )\times \tilde{{\mathcal {P}}}_*(\Sigma ^2)$$,
(ii) *I is a good rate function and*
(iii) ${{\mathfrak {H}}_2}(\omega \,\Vert \, \eta _1)\ge 0$*with equality if and only if*$\omega =\Delta (d)C_d\eta _1\otimes \eta _1 .$

#### *Proof*

(i) Suppose that $\omega \not \ll \Delta (d) C_d\eta _1\otimes \eta _1 .$ Then, there exists $a_0,b_0 \in \Sigma$ with $C\eta _1\otimes \eta _1 (a_0,b_0)=0$ and $\omega (a_0,b_0)>0.$ Define $\hat{g}{:}\; \Sigma ^2\rightarrow \mathbb {R}$ by

$$\begin{aligned} \hat{g}(a,b)=\log \left[ K(\mathbbm {1}_{(a_0,b_0)}(a,b)+\mathbbm {1}_{(b_0,a_0)}(a,b))+1\right] , \quad \text{for } a,b\in \Sigma \text{ and } K>0. \end{aligned}$$

For this choice of $\hat{g}$ and $f=0$ we have

$$\begin{aligned}&\sum _{a\in \Sigma }\left( f(a)-U_{f}\right) \eta _{1}(a) +\sum _{a,b\in \Sigma } \frac{1}{2} \hat{g}(a,b)\omega (a,b)+\sum _{a,b\in \Sigma }\frac{\Delta (d)}{2}(1-e^{\hat{g}(a,b)})C_d(a,b)\eta _{1}(a) \eta _{1}(b) \\&\quad \ge \frac{\Delta (d)}{2} \log (K+1)\omega (a_{0},b_{0}) \rightarrow \infty , \quad \text{ for } K\uparrow \infty . \end{aligned}$$

Now suppose that $\omega \ll C\eta _1\otimes \eta _1 .$ We have

$$\begin{aligned} \begin{aligned} \hat{I}(\eta _1,\omega )&= \sup _{f\in {\mathcal {C}}_{1}} \left\{ \sum _{a\in \Sigma }\left( f(a)-\log \sum _{a\in \Sigma }e^{{f}(a)}\nu (a)\right) \, \eta _{1}(a) \right\} \\&\quad + \frac{\Delta (d)}{2}\sum _{a,b\in \Sigma } C_d(a,b)\eta _1(a)\eta _{1}(b) \\&\quad + \frac{1}{2} \sup _{g\in {\mathcal {C}}_{2}} \left\{ \sum _{a,b\in \Sigma }g(a,b)\omega (a,b)- \Delta (d)\sum _{a,b\in \Sigma } e^{g(a,b)} C_d(a,b)\eta _1(a)\eta _{1}(b) \right\} . \end{aligned} \end{aligned}$$

By the variational characterization of relative entropy, the first term equals $H(\eta _1 \, \Vert \, \nu )$. By the substitution $h=\Delta (d)e^{g} \, \frac{C_d \eta _1 \otimes \eta _1}{\omega }$ the last term equals

$$\begin{aligned} \begin{aligned}&\sup _{\genfrac{}{}{0.0pt}{}{h\in {\mathcal {C}}_{2}}{h \ge 0}} \sum _{a,b\in \Sigma } \left[ \log \left( h(a,b) \frac{\omega (a,b)}{\Delta (d)C_d(a,b)\eta _1(a) \eta _{1}(b) }\right) -h(a,b) \right] \, \omega (a,b) \\&\quad = \sup _{\genfrac{}{}{0.0pt}{}{h\in {\mathcal {C}}_{2}}{h \ge 0}} \sum _{a,b\in \Sigma } \left( \log h(a,b) - h(a,b) \right) \, \omega (a,b) + \sum _{a,b\in \Sigma } \log \left( \frac{\omega (a,b)}{\Delta (d)C_d(a,b) \eta _1(a) \eta _1(b)} \right) \, \omega (a,b) \\&\quad = - \Vert \omega \Vert + H(\omega \, \Vert \,\Delta (d) C_d \eta _1\otimes \eta _1 ), \end{aligned} \end{aligned}$$

where we have used $\sup _{x>0} \log x - x = -1$ in the last step. This yields that $\hat{I}(\eta _1,\omega )={I}(\eta _1,\omega )$.

(ii) Recall from (3) and the definition of ${{{\mathfrak {H}}}_2}$ that $I(\eta _1, \omega )=H(\omega \,\Vert \,\nu )+ \frac{1}{2}\, H\left( \omega \,\Vert \,\Delta (d)C_d\eta _1\otimes \eta _1 \right) +\frac{\Delta (d)}{2}\, \Vert C_d\eta _1\otimes \eta _1 \Vert - \frac{1}{2}\, \Vert \omega \Vert$. All summands are continuous in $\eta _1, \omega$ and thus *I* is a rate function. Moreover, for all $\alpha <\infty$, the level sets $\{I \le \alpha \}$ are contained in the bounded set $\{(\eta _1, \omega )\in {\mathcal {P}}(\Sigma )\times \tilde{{\mathcal {P}}}_*(\Sigma ^2){:}\; \,{{\mathfrak {H}}_2}(\omega \,\Vert \,\eta _1)\le \alpha \}$ and are therefore compact. Consequently, *I* is a good rate function.

(iii) Consider the nonnegative function $\xi (x)=x\log x-x+1$, for $x> 0$, $\xi (0)=1$, which has its only root in $x=1$. Note that

10 $$\begin{aligned} {{\mathfrak {H}}_2}(\omega \,\Vert \,\eta _1)= \left\{ \begin{array}{ll}\int \xi \circ g\,\,d(\Delta (d)C_d\omega \otimes \omega ) &{}\quad \text{ if } g:=\frac{d\omega }{d(\Delta (d)C_d\eta _1\otimes \eta _1 )}\ge 0 \text{ exists, } \\ \infty &{} \quad \text{ otherwise. } \end{array}\right. \end{aligned}$$

Hence ${{\mathfrak {H}}_2}(\omega \,\Vert \,\eta _1)\ge 0$, and if $\omega =\Delta (d)C_d\eta _1\otimes \eta _1 ,$ then $\xi \left( \frac{d\omega }{d(\Delta (d)C_d\eta _1\otimes \eta _1 )}\right) =\xi (1)=0$ and so ${{\mathfrak {H}}_2}(\Delta (d)C_d\eta _1\otimes \eta _1 \,\Vert \,\omega )=0$. Conversely, if ${{\mathfrak {H}}_2}(\omega \,\Vert \,\omega )=0$, then $\omega (a,b)>0$ implies $C_d\eta _1\otimes \eta _1 (a,b)>0$, which then implies $\xi \circ g(a,b)=0$ and further $g(a,b)=1$. Hence $\omega =\Delta (d)C_d\eta _1\otimes \eta _1,$ which completes the proof of (iii). $\square$

### Proof of the lower bound in Theorem 4

We obtain the lower bound of Theorem 4 from the upper bound as follows:

#### **Lemma 5**

*For every open set*$O\subset {\mathcal {P}}(\Sigma )\times \tilde{{\mathcal {P}}}_*(\Sigma ^2),$*we have*

$$\begin{aligned} \liminf _{n\rightarrow \infty }\frac{1}{n}\log \mathbb {P}\left\{ \left( {\mathcal {L}}_{{\mathcal {G}}}^1,{\mathcal {L}}_{{\mathcal {G}}}^2\right) \in O\right\} \ge -\inf _{(\eta _1, \omega )\in O}I(\eta _1, \omega ). \end{aligned}$$

#### *Proof*

Suppose $(\eta _1, \omega )\in O,$ with $\omega \ll \Delta (d)C_d\eta _1\otimes \eta _1$. Define $\tilde{f}_{\omega }{:}\;\Sigma \rightarrow \mathbb {R}$ by

$$\begin{aligned} \begin{aligned} \tilde{f}_{\omega }(a)=\left\{ \begin{array}{ll}\log \frac{\eta _1(a)}{\nu (a)}, &{}\quad \text{ if } \eta _1(a)> 0,\\ 0, &{}\quad \text{ otherwise. } \end{array}\right. \end{aligned} \end{aligned}$$

and $$\tilde{g}_{\omega }{:}\;\Sigma ^2\rightarrow \mathbb {R}$$ by

$$\begin{aligned} \begin{aligned} \tilde{g}_{\omega }(a,b)=\left\{ \begin{array}{ll}\log \frac{\omega (a,b)}{\Delta (d)C_d(a,b)\eta _1(a)\eta _1(b)},&{}\quad \text{ if } \omega (a,b)>0, \\ 0, &{}\quad \text{ otherwise. } \end{array}\right. \end{aligned} \end{aligned}$$

In addition, we let $\tilde{\beta }_{\omega }(a,b)=\Delta (d)C_d(a,b)(1-e^{{\tilde{g}}_{\omega }(a,b)})$ and note that $\tilde{\beta }_{\omega }(a,b)=\lim _{n\rightarrow \infty }\tilde{h}_{\omega , n}(a,b),$ for all $a,b\in \Sigma$ where

$$\begin{aligned} \tilde{h}_{\omega , n}(a,b)=\log \left[ \left( 1-F(r_n(a,b))+F(r_n(a,b))e^{\tilde{g}_{\omega }(a,b)}\right) ^{-n}\right] . \end{aligned}$$

Choose $B_{\eta _1}^{1} ,B_{\omega }^{2}$ open neighbourhoods of $\eta _1, \omega ,$ such that $B_{\eta _1}^{1}, \times B_{\omega }^{2}\subset O$ and for all $(\tilde{\omega },\tilde{\omega })\in B_{\eta _1}^{1}\times B_{\omega }^{2}$

$$\begin{aligned} \langle \tilde{f}_{\omega },\eta _1\rangle +\frac{1}{2}\, \langle \tilde{g}_{\omega },\omega \rangle + \frac{1}{2}\, \langle \tilde{\beta }_{\omega },\eta _1\otimes \eta _1 \rangle -\varepsilon \le \langle \tilde{f}_{\omega },\tilde{\eta }_1\rangle + \frac{1}{2}\, \langle \tilde{g}_{\omega },\tilde{\omega }\rangle + \frac{1}{2}\, \langle \tilde{\beta }_{\omega },\tilde{\eta }_1\otimes \tilde{\eta }_1\rangle . \end{aligned}$$

We now use $\tilde{\mathbb {P}},$ the probability measure obtained by transforming $\mathbb {P}$ using the functions $\tilde{f}_{\omega }$, $\tilde{g}_{\omega }$. Note that the colour law in the transformed measure is now $\eta _1$, and the connectivity radii $\tilde{r}_n(a,b)$ satisfy

$$\begin{aligned} n \, \tilde{r}_n^d(a,b) \rightarrow {\omega (a,b)}/(\eta _1(a)\eta _1(b)) =: \tilde{C}_d(a,b),\quad \text{ as } n\rightarrow \infty . \end{aligned}$$

Using (5), we obtain

$$\begin{aligned}&\mathbb {P}\left \{\left( {\mathcal {L}}_{{\mathcal {G}}}^1,{\mathcal {L}}_{{\mathcal {G}}}^2\right) \in O\right \}\ge \tilde{\mathbb {E}}\left \{\frac{d\mathbb {P}}{d\tilde{\mathbb {P}}}({\mathcal {G}})\mathbbm {1}_{ \left\{ \left( {\mathcal {L}}_{{\mathcal {G}}}^1,{\mathcal {L}}_{{\mathcal {G}}}^2\right) \in B_{\eta _1}^{1} \times B_{\omega }^{2}\right\} }\right \}\\&\quad =\tilde{\mathbb {E}}\left\{ \prod _{u\in V}e^{-\tilde{f}_{\omega }(\sigma (x_u))}\prod _{(u,v)\in E}e^{-\tilde{g}_\omega (\sigma (x_u),\sigma (x_v))}\prod _{(u,v)\in {\mathcal {E}}}e^{-\frac{1}{n} \, \tilde{h}_{\omega , n}(\sigma (x_u),\sigma (x_v))} \mathbbm {1}_{\left\{ \left( {\mathcal {L}}_{{\mathcal {G}}}^1,{\mathcal {L}}_{{\mathcal {G}}}^2\right) \in B_{\eta _1}^{1} \times B_{\omega }^{2}\right\} }\right\} \\&\quad =\tilde{\mathbb {E}}\left\{ e^{-n\left\langle {\mathcal {L}}_{{\mathcal {G}}}^1, \tilde{f}_{\omega }\right\rangle -n\frac{1}{2}\, \left\langle {\mathcal {L}}_{{\mathcal {G}}}^2, \tilde{g}_{\omega }\right\rangle -n\frac{1}{2}\,\left\langle {\mathcal {L}}_{{\mathcal {G}}}^1\otimes {\mathcal {L}}_{{\mathcal {G}}}^1, {\tilde{g}}_{\omega }\right\rangle +\frac{1}{2}\,\left\langle L_{\Delta }^{1},\tilde{h}_{\omega , n}\right\rangle }\times \mathbbm {1}_{\left\{ \left( {\mathcal {L}}_{{\mathcal {G}}}^1,{\mathcal {L}}_{{\mathcal {G}}}^2\right) \in B_{\eta _1}^{1} \times B_{\omega }^{2}\right\} }\right\} \\&\quad \ge \exp \left( -n\langle \tilde{f}_{\omega },\omega \rangle -n\frac{1}{2} \langle \tilde{g}_{\omega },\omega \rangle -n \frac{1}{2} \langle \tilde{\beta }_\omega ,\eta _1\otimes \eta _1 \rangle +m-n\varepsilon \right) \times \tilde{\mathbb {P}}\left\{ \left( {\mathcal {L}}_{{\mathcal {G}}}^1,{\mathcal {L}}_{{\mathcal {G}}}^2\right) \in B_{\eta _1}^{1} \times B_{\omega }^{2}\right\} , \end{aligned}$$

where $m:=0 \wedge \min _{a\in \Sigma }\tilde{\beta }(a,a).$ Therefore, by (6), we have

$$\begin{aligned}&\liminf _{n\rightarrow \infty }\frac{1}{n}\log \mathbb {P}\left\{ \left( {\mathcal {L}}_{{\mathcal {G}}}^1,{\mathcal {L}}_{{\mathcal {G}}}^2\right) \in O\right\} \\&\quad \ge -\langle \tilde{f}_{\omega },\omega \rangle -\frac{1}{2} \, \langle \tilde{g}_{\omega },\omega \rangle -\frac{1}{2}\, \langle {\tilde{{\beta }}_{\omega }}, \eta _1\otimes \eta _1 \rangle -\varepsilon +\liminf _{n\rightarrow \infty }\frac{1}{n}\log \tilde{\mathbb {P}} \left\{ \left( {\mathcal {L}}_{{\mathcal {G}}}^1,{\mathcal {L}}_{{\mathcal {G}}}^2\right) \in B_{\eta _1}^{1} \times B_{\omega }^{2}\right\} . \end{aligned}$$

The result follows once we prove that

11 $$\begin{aligned} \liminf _{n\rightarrow \infty }\frac{1}{n}\log \tilde{\mathbb {P}}\left\{ \left( {\mathcal {L}}_{{\mathcal {G}}}^1,{\mathcal {L}}_{{\mathcal {G}}}^2\right) \in B_{\eta _1}^{1} \times B_{\omega }^{2}\right\} = 0. \end{aligned}$$

We use the upper bound (but now with the law $\mathbb {P}$ replaced by $\tilde{\mathbb {P}}$) to prove (11). Then we obtain

$$\begin{aligned} \limsup _{n\rightarrow \infty }\frac{1}{n}\log \tilde{\mathbb {P}}\left\{ \left( {\mathcal {L}}_{{\mathcal {G}}}^1,{\mathcal {L}}_{{\mathcal {G}}}^2\right) \in \left(B_{\eta }^{1} \times B_{\omega }^{2}\right)^{c} \right\}&\le -\inf _{(\tilde{\rho },\tilde{\omega })\in \tilde{F}} \tilde{I}(\tilde{\rho },\tilde{\omega }), \end{aligned}$$

where $\tilde{F}=(B_{\eta _1}^{1} \times B_{\omega }^{2})^{c}$ and $\tilde{I}(\tilde{\rho }, \tilde{\omega }):= H(\tilde{\omega } \, \Vert \, \omega ) + \frac{1}{2} {{\mathfrak {H}}_{2}}(\tilde{\omega }\,\Vert \, \tilde{\rho })$. It therefore suffices to show that the infimum is positive. Suppose for contradiction that there exists a sequence $(\tilde{\rho }_n,\tilde{\omega }_n)\in \tilde{F}$ with $\tilde{I}(\tilde{\rho }_n,\tilde{\omega }_n)\downarrow 0.$ Then, because $\tilde{I}$ is a good rate function and its level sets are compact, and by lower semi-continuity of the mapping $(\tilde{\rho },\tilde{\omega })\mapsto \tilde{I}(\tilde{\rho },\tilde{\omega })$, we can construct a limit point $(\tilde{\rho },\tilde{\omega })\in \tilde{F}$ with $\tilde{I}(\tilde{\rho },\tilde{\omega })=0$. By Lemma 4 this implies $H(\tilde{\rho }\,\Vert \,\eta _1)=0$ and ${{\mathfrak {H}}_2}(\tilde{\omega }\,\Vert \,\eta _1)=0$, hence $\tilde{\rho }=\eta _1,$ and $\tilde{\omega }=\tilde{C}_d\eta _1\otimes \eta _1=\omega$ contradicting $(\tilde{\rho },\tilde{\omega })\in \tilde{F}$. $\square$

## Proof of Theorem 1

For any $n\in \mathbb {N}$ we define

$$\begin{aligned} \begin{aligned} {\mathcal {P}}_n(\Sigma )&:= \left\{ \rho \in {\mathcal {P}}(\Sigma ) {:}\; n\rho (a) \in \mathbb {N}\quad \text{for } \text{ all } a\in \Sigma \right\} ,\\ \tilde{{\mathcal {P}}}_n(\Sigma \times \Sigma )&:= \left\{ \omega \in \tilde{{\mathcal {P}}}_*(\Sigma \times \Sigma ) {:}\; \frac{n}{1+\mathbbm {1}\{a=b\}}\,\omega (a,b) \in \mathbb {N} \quad \text{for } \text{ all } a,b\in \Sigma \right\} . \end{aligned} \end{aligned}$$

We denote by $\Theta _n:={\mathcal {P}}_n(\Sigma )\times \tilde{{\mathcal {P}}}_n(\Sigma \times \Sigma )$ and $\Theta :={\mathcal {P}}(\Sigma )\times \tilde{{\mathcal {P}}}_*(\Sigma \times \Sigma )$. With

$$\begin{aligned} \begin{aligned} P_{(\rho _n, \omega _n)}^{(n)}(\eta _n)&:= \mathbb {P}\left\{ {\mathcal {M}}_{{\mathcal {G}}}=\eta _n \, \big | \, {\mathcal {H}}({\mathcal {M}}_{{\mathcal {G}}})=(\rho _n,\omega _n)\right\} ,\\ P^{(n)}(\rho _n,\omega _n)&:= \mathbb {P}\left\{ \left( {\mathcal {L}}_{{\mathcal {G}}}^1,{\mathcal {L}}_{{\mathcal {G}}}^2\right) =(\rho _n,\omega _n)\right\} \end{aligned} \end{aligned}$$

the joint distribution of ${\mathcal {L}}_{{\mathcal {G}}}^1, {\mathcal {L}}_{{\mathcal {G}}}^2$ and ${\mathcal {M}}_{{\mathcal {G}}}$ is the mixture of $P_{(\rho _n, \omega _n)}^{(n)}$ with $P^{(n)}(\rho _n,\omega _n)$ defined as

12 $$\begin{aligned} d\tilde{P}^n(\rho _n, \omega _n, \eta _n):= dP_{(\rho _n, \omega _n)}^{(n)}(\eta _n)\, dP^{(n)}(\rho _n, \omega _n).\, \end{aligned}$$

Biggins (2004, Theorem 5(b)) gives criteria for the validity of large deviation principles for the mixtures and for the goodness of the rate function if individual large deviation principles are known. The following three lemmas ensure validity of these conditions.

We recall from Lemma 6 that the family of measures $({P}^n {:}\; n\in \mathbb {N})$ is exponentially tight on $\Theta$

### **Lemma 6**

(Doku-Amponsah and Mörters 2010) *The family of measures*$(\tilde{P}^n {:}\; n\in \mathbb {N})$*is exponentially tight on*$\Theta \times {\mathcal {P}}(\Sigma \times \mathbb {N}).$

Define the function

$$\begin{aligned} \tilde{J}{:}\;{\Theta }\times {\mathcal {P}}(\Sigma \times \mathbb {N})\rightarrow [0,\infty ], \quad \tilde{J}((\eta _1,\omega ),\,\eta )=\tilde{J}_{(\eta _1,\omega )}(\eta ), \end{aligned}$$

where

13 $$\begin{aligned} \tilde{J}_{(\eta _1,\omega )}(\eta )=\left\{ \begin{array}{ll}H\left(\eta \,\Vert \,Q_{poi}^{(\omega , \eta )}\right) &{} \quad \text{ if } (\omega , \eta ) \text{ is } \text{ consistent } \text{ and } \eta _1=\omega _2\\ \infty &{} \quad \text{ otherwise. } \end{array}\right. \end{aligned}$$

### **Lemma 7**

(Doku-Amponsah and Mörters 2010) $\tilde{J}$*is lower semi-continuous.*

By Biggins (2004, Theorem 5(b)) the two previous lemmas and the large deviation principles we have established Theorem 2.2 and Doku-Amponsah (2015, Theorem 2.1) ensure that under $(\tilde{P}^n)$ the random variables $(\rho _n, \omega _n, \eta _n)$ satisfy a large deviation principle on ${\mathcal {P}}(\Sigma ) \times \tilde{{\mathcal {P}}}_*(\Sigma \times \Sigma ) \times {\mathcal {P}}(\Sigma \times \mathbb {N})$ with good rate function

$$\begin{aligned} \hat{J}(\eta _1, \omega , \eta )= \left\{ \begin{array}{ll} H(\eta _1\,\Vert \,\nu ) + \frac{1}{2}\,{{\mathfrak {H}}}_2(\omega \,\Vert \,\Sigma ) + H\left(\eta \,\Vert Q_{poi}^{(\omega ,\eta )}\right) , &{} \quad \text{ if } (\omega ,\eta ) \text{ is } \text{ consistent } \text{ and } \eta _1=\omega _2, \\ \infty , &{}\quad \text{ otherwise. } \\ \end{array}\right. \end{aligned}$$

By projection onto the last two components we obtain the large deviation principle as stated in Theorem 1 from the contraction principle, see e.g. Dembo et al. (2005, Theorem 4.2.1) .

## Proof of Corollaries 2, 3, and 5

We derive the theorems from Theorem 1 by applying the contraction principle, see e.g. Dembo and Zeitouni (1998, Theorem 4.2.1). In fact Theorem 1 and the contraction principle imply a large deviation principle for *D*. It just remains to simplify the rate functions.

### Proof of Theorem 2

Note that, in the case of an uncoloured RGG graphs, the function *C* degenerates to a constant *c*, ${\mathcal {L}}_{{\mathcal {G}}}^2=|E|/n\in [0,\infty )$ and ${\mathcal {M}}_{{\mathcal {G}}}=D\in {\mathcal {P}}(\mathbb {N}\cup \{0\})$. Theorem 1 and the contraction principle imply a large deviation principle for *D* with good rate function

$$\begin{aligned} \begin{aligned} \lambda _2(\delta )&=\inf \{J(x,\delta ) {:}\; x\ge 0 \} =\inf \left\{ H(\delta \,\Vert \,q_{x})+\frac{1}{2} x\log x -\frac{1}{2} x\log \Delta (d)c+ \frac{1}{2}\,\Delta (d)c-\frac{1}{2} x {:}\; \langle \delta \rangle \le x\,\right\} , \end{aligned} \end{aligned}$$

which is to be understood as infinity if $\langle d\rangle$ is infinite. We denote by $\lambda ^{x}(\delta )$ the expression inside the infimum. For any $\varepsilon >0$, we have

$$\begin{aligned} \begin{aligned} \lambda _2^{\langle \delta \rangle +\varepsilon }(\delta )-\lambda _2^{\langle \delta \rangle }(\delta )&=\frac{\varepsilon }{2} +\frac{\langle \delta \rangle -\varepsilon }{2}\log \frac{\langle \delta \rangle }{\langle \delta \rangle +\varepsilon }+\frac{\varepsilon }{2}\log \frac{\langle \delta \rangle }{\Delta (d)c} \ge \frac{\varepsilon }{2} +\frac{\langle \delta \rangle -\varepsilon }{2}\, \left( \frac{-\varepsilon }{\langle \delta \rangle }\right) +\frac{\varepsilon }{2}\log \frac{\langle \delta \rangle }{\Delta (d)c} >0, \end{aligned} \end{aligned}$$

so that the minimum is attained at $x=\Delta (d)\langle \delta \rangle$.

### Proof of Corollary 3

Corollary 3 follows from Theorem 2 and the contraction principle applied to the continuous linear map $G{:}\;{\mathcal {P}}(\mathbb {N}\cup \{0\})\rightarrow [0,\,1]$ defined by $G(\delta )=\delta (0).$ Thus, Theorem 2 implies the large deviation principle for $G(D)=W$ with the good rate function $\xi _2(y)=\inf \{\lambda _2(\delta ) {:}\; \delta (0)=y, \langle \delta \rangle < \infty \}.$ We recall the definition of $\lambda _2^{x}$ and observe that $\xi _2(y)$ can be expressed as

$$\begin{aligned} \xi _2(y)=\inf _{b\ge 0}\inf _{\genfrac{}{}{0.0pt}{}{d\in {\mathcal {P}}(\mathbb {N}\cup \{0\})}{\delta (0)=y,\, \Delta (d)c\langle \delta \rangle =b^2}} \left\{ \frac{1}{2}c+y\log y+\frac{b^2}{2\Delta (d)c} +\sum _{k=1}^{\infty }\delta (k)\log \frac{\delta (k)}{q_{b}(k)}-b(1-y)\right\} . \end{aligned}$$

Now, using Jensen’s inequality, we have that

14 $$\begin{aligned} \sum _{k=1}^{\infty }\delta (k)\log \frac{\delta (k)}{q_{b}(k)}\ge (1-y)\log \frac{(1-y)}{(1-e^{-b})}, \end{aligned}$$

with equality if $\delta (k)=\frac{(1-y)}{(1-e^{-b})}q_{b}(k),$ for all $k\in \mathbb {N}.$ Therefore, we have the inequality

$$\begin{aligned} \inf \{\lambda _2(\delta ) {:}\; \delta (0)=y, \langle \delta \rangle <\infty \} \ge \inf \left\{ \frac{1}{2}c+y\log y+\frac{b^2}{2\Delta (d)c} +(1-y)\log \frac{(1-y)}{(1-e^{-b})} -b(1-y) {:}\; b\ge 0 \right\} . \end{aligned}$$

Let $y\in [0,\,1].$ Then, the equation $a(1-e^{-a})=\Delta (d)c(1-y)$ has a unique positive solution. Elementary calculus shows that the global minimum of $b\mapsto \frac{1}{2}\Delta (d)c+y\log y+\frac{b^2}{2\Delta (d)c} +(1-y)\log \frac{(1-y)}{(1-e^{-b})} -b(1-y)$ on $(0,\infty )$ is attained at the value $b=a$, where *a* is the positive solution of our equation. We obtain the form of $\xi$ in Corollary 3 by observing that

$$\begin{aligned} \frac{a(y)^2+(\Delta (d)c)^2 -2\Delta (d)ca(y)\left( 1-y\right) }{2\Delta (d)c}=\frac{\Delta (d)cy}{2}\left( 2-y\right) +\frac{1}{2\Delta (d)c}\left( a(y)-\Delta (d)c(1-y)\right) ^2. \end{aligned}$$

### Proof of Corollary 5

We define the continuous linear map $W{:}\;{\mathcal {P}}(\Sigma )\times \tilde{{\mathcal {P}}}_*(\Sigma ^2) \rightarrow [0,\infty )$ by $W(\eta _1,{\omega })=\frac{1}{2}\Vert {\omega }\Vert ,$ and infer from Theorem 4 and the contraction principle that $W({\mathcal {L}}_{{\mathcal {G}}}^1,{\mathcal {L}}_{{\mathcal {G}}}^2)=|E|/n$ satisfies a large deviation principle in $[0,\infty )$ with the good rate function

$$\begin{aligned} \zeta (y)=\inf \left\{ I(\eta _1,{\omega }) {:}\; W(\eta _1,{\omega })=y\right\} . \end{aligned}$$

To obtain the form of the rate in the corollary, the infimum is reformulated as unconstrained optimization problem (by normalising $\omega$)

15 $$\begin{aligned} \inf _{\genfrac{}{}{0.0pt}{}{\omega \in {\mathcal {P}}_*(\Sigma ^2)}{\eta _1\in {\mathcal {P}}(\Sigma )}} \left\{ H(\eta _1\,\Vert \,\nu )+yH(\omega \,\Vert \,\Delta (d)C\eta _1\otimes \eta _1 )+y\log 2y+ \frac{\Delta (d)}{2}\, \Vert C \omega \otimes \omega \Vert -y\right\} . \end{aligned}$$

By Jensen’s inequality $H(\omega \,\Vert \,\Delta (d)C\eta _1\otimes \eta _1 )\ge -\log \Vert \Delta (d)C\eta _1\otimes \eta _1 \Vert ,$ with equality if $\omega =\frac{C\eta _1\otimes \eta _1 }{\Vert C\eta _1\otimes \eta _1 \Vert },$ and hence, by symmetry of *C* we have

$$\begin{aligned}&\min _{\omega \in {\mathcal {P}}_*(\Sigma ^2)} \left\{ H(\eta _1\,\Vert \,\nu )+yH(\omega \,\Vert \,\Delta (d)C\eta _1\otimes \eta _1 ) +y\log 2y+\frac{\Delta (d)}{2}\, \Vert C \eta _1\otimes \eta _1 \Vert -y\right\} \\&\quad = H(\eta _1\,\Vert \,\nu ) -y\log \Vert \Delta (d)C \eta _1\otimes \eta _1 \Vert +y\log 2y + \frac{\Delta (d)}{2}\, \Vert C \eta _1\otimes \eta _1 \Vert -y. \end{aligned}$$

The form given in Corollary 5 follows by defining

$$\begin{aligned} y=\frac{1}{2}\Delta (d)\sum _{a,b\in \Sigma } C_d(a,b)\eta _1(a)\eta _1(b). \end{aligned}$$

## Conclusion

In this work, we have proved joint large deviation principle for the empirical pair measure and empirical locality measure of the near intermediate CGRG models. From this result we have obtained asymptotic results about useful graph quantities such as number of edges per vertex, the degree distribution and the proportion of isolated vertices for the near intermediate CGRG models. The rate functions of all these large deviation principles compared very well with the rate functions of the results for coloured random graph models by Doku-Amponsah and Mörters (2010), with some extra terms accounting for the geometric effect in the CGRG models. An important future research direction is to formulate and prove an Asymptotic Equipartition Property for Networked Data Structures Modelled as the CGRG, and then a possible Coding or Approximate Pattern Matching Algorithms for such Networks. One could also investigate the Statistical Mechanics on the CGRG.
